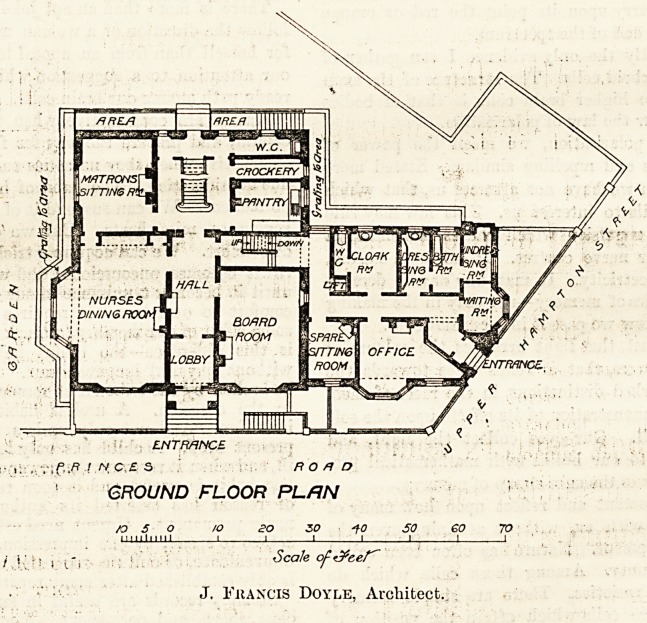# The Liverpool Central Home for District Nurses

**Published:** 1901-03-02

**Authors:** 


### The Institutional Workshop.

### THE LIVERPOOL CENTRAL HOME FOR DISTRICT NURSES.

The Liverpool Central Home for District Nurses, of which we to-day give the plans, was opened in October last, having been presented to the city of Liverpool by Mr. B. W. Levy and the "David Lewis" Trust to assist in promoting and extending the work of nursing the sick

FIRST FLOOR PLAN.

SECOND FLOOR PLAN.

GROUND FLOOR PLAN

10 5 0 10 20 30 40 50 60 70

Scale of Feet

J. Francis Doyle, Architect.

poor in their own homes. This building, which is situated at the corner of Prince's Road and Parliament Street, will form the principal of four homes in which the thirty-four Queen's Nurses at present working in various districts in the city will reside, each home being presided over by a resident matron. This being the central home it has been necessary to provide a board-room in addition to the accommodation for the matron, nurses, and staff.

The principal entrance is approached by a few steps and opens into a lobby and hall 9 feet wide which leads to a handsome staircase extending from the basement to the first floor. On the ground floor are the board-room, the nurses' dining-room, a handsome room 28 feet long with two windows one of which is a bay, the matron's sitting-room, a pantry, &c., and going down a few steps one enters a wing having a separate entrance, with an office, a spare sitting-room, a cloak-room, &c., and a bath-room with an undressing as well as a dressing-room attached, the object being to enable a nurse who has been attending an infectious case to change and bathe before mixing with the rest of the staff. We cannot but think that this portion of the building might usefully have been arranged in a somewhat different manner. It will be seen by reference to the plan annexed that the nurse whom one may regard as infected cannot get to her unrobing-room without passing through both the dressing-room and the bath-room; an unsatisfactory arrangement, and one not to compare with the plan usually adopted at isolation hospitals for the bathing of the nurses and the changing of their clothes before going out when they have a holiday.

On the first floor there is a nurses' sitting-room, a bath-room, an assistant matron's bed and sitting-room combined, and the matron's sitting-room and her bedroom, while in what we call the annexe, down a few steps, there are five bedrooms and a bath-room and w.c.'s, &c. The bedrooms vary in size, measuring from about 12 by 12 feet to 11 by 9 feet, and each is provided with a fireplace. We should have thought much better of the planning of this part of the building if, instead of heaping the closets in the corner, they could have been brought out to the other end of the corridor, so as to provide some sort of aërial "cut off" between them and the bedrooms. The same remark applies to the other floors. Nor do we see why the assistant-matron's bed-sitting-room is made to open into a bath-room and lavatory. This doorway should certainly be filled up.

The second floor contains bedrooms and cubicles, and a sick-block containing two bedrooms, a bath-room with a fireplace, and a w.c. This sick-block is a very useful addition to any institution of the kind. It is entirely separated from the rest of the building by a straight wall through which there is only a single doorway—all of which is good. We cannot but think, however, that the position of this door, opening as it does into an apparently unventilated corridor in the midst of the servants' bedrooms, is badly placed. If it is thought desirable to have such a completely isolated sick-block it must be well to have the door leading to it placed as near as possible to the fresh air.

The mezzanine floor of the annexe follows the same plan as the first floor. It is always difficult to adapt a building to a new purpose, and probably some of the points which we have criticised may have been due to the structural difficulties involved. With these exceptions the building seems to have been very carefully arranged, and certainly it is well suited for the purpose for which it is intended. The whole of the work of adapting and adding to the building has been carried out from the design and under the supervision of Mr. J. Francis Doyle, architect, and Mr. Isaac Dilworth was the contractor for the building.

## Figures and Tables

**Figure f1:**
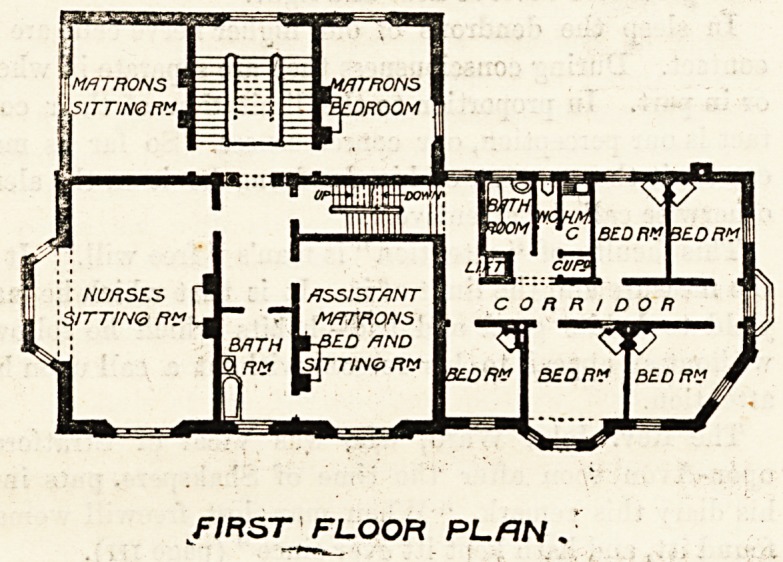


**Figure f2:**
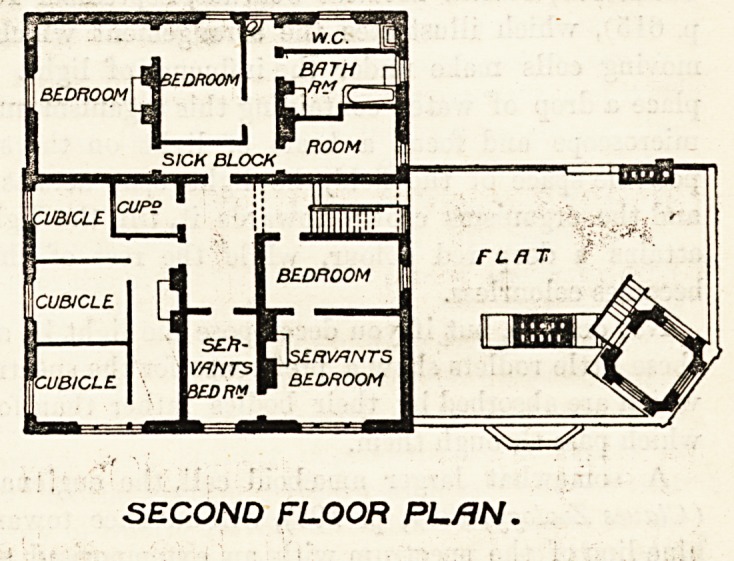


**Figure f3:**